# Adoption of a Contact Tracing App for Containing COVID-19: A Health Belief Model Approach

**DOI:** 10.2196/20572

**Published:** 2020-09-01

**Authors:** Michel Walrave, Cato Waeterloos, Koen Ponnet

**Affiliations:** 1 Research Group MIOS Department of Communication Studies, Faculty of Social Sciences University of Antwerp Antwerp Belgium; 2 Research Group IMEC-MICT Department of Communication Sciences, Faculty of Political and Social Sciences Ghent University Ghent Belgium

**Keywords:** COVID-19, SARS-CoV-2, health belief model, contact tracing, proximity tracing, privacy

## Abstract

**Background:**

To track and reduce the spread of COVID-19, apps have been developed to identify contact with individuals infected with SARS-CoV-2 and warn those who are at risk of having contracted the virus. However, the effectiveness of these apps depends highly on their uptake by the general population.

**Objective:**

The present study investigated factors influencing app use intention, based on the health belief model. In addition, associations with respondents’ level of news consumption and their health condition were investigated.

**Methods:**

A survey was administered in Flanders, Belgium, to 1500 respondents, aged 18 to 64 years. Structural equation modeling was used to investigate relationships across the model’s constructs.

**Results:**

In total, 48.70% (n=730) of respondents indicated that they intend to use a COVID-19 tracing app. The most important predictor was the perceived benefits of the app, followed by self-efficacy and perceived barriers. Perceived severity and perceived susceptibility were not related to app uptake intention. Moreover, cues to action (ie, individuals’ exposure to [digital] media content) were positively associated with app use intention. As the respondents’ age increased, their perceived benefits and self-efficacy for app usage decreased.

**Conclusions:**

Initiatives to stimulate the uptake of contact tracing apps should enhance perceived benefits and self-efficacy. A perceived barrier for some potential users is privacy concerns. Therefore, when developing and launching an app, clarification on how individuals’ privacy will be protected is needed. To sustain perceived benefits in the long run, supplementary options could be integrated to inform and assist users.

## Introduction

The rapid spread of COVID-19 has led to numerous efforts to contain the pandemic as scientists endeavor to develop potential vaccinations. While policy makers have implemented several measures, it has been proposed that technologies be integrated into countries’ deconfinement strategies. To reduce the risk of spreading SARS-CoV-2 when exiting lockdown measures, several apps have been developed. At the core of these apps is contact tracing. Through contact tracing, the potential transmission routes of a virus in the population can be assessed to isolate and assist individuals who may have been in contact with someone with COVID-19 [1]. By using an app that traces contact with COVID-19–infected individuals and offers advice on how to prevent infection, citizens can help limit the spread of the virus.

However, the effectiveness of this app depends on uptake by the population [1]. Therefore, this study investigated factors that can influence citizens’ willingness to use an app that traces contact with COVID-19–diagnosed individuals and notifies app users of this contact, without revealing the identity of the diagnosed app user(s) or where this contact occurred. This proximity tracing is made possible by the exchange of random identification codes between smartphones that are running the app and are in each other’s proximity. The smartphones save this list of codes for a period of time (eg, the incubation period of the virus). When a smartphone user is diagnosed with the virus, they can upload anonymized data to the app’s server, with the explicit permission of the user and approval of a health professional. App users who have been in the proximity of the infected app user during the incubation period of the virus will be informed that they have been in contact with an individual who has been infected with COVID-19 and therefore might be at risk of having contracted the virus. This notification to at-risk individuals can further advise users on what steps to undertake (eg, getting tested, self-isolation).

A number of countries have integrated this kind of tracing app into their deconfinement plans or are presently discussing this option [2,3]. Research has concentrated on contact tracing and symptom tracking systems [1,4-6] as well as the association between app usage and the epidemiological spread of the virus [7]. Some studies have focused on the differences between apps implemented in several countries [3], while others have analyzed the legal or ethical aspects (eg, data protection) [2,8,9]. Questions still remain about the factors that influence citizens’ uptake of COVID-19 contact tracing apps. Insight into these factors provides developers and policy makers information on aspects that need to be taken into account when launching an app and stimulating app uptake.

The aim of this study is to investigate which factors influence individuals’ intention to use a COVID-19 app by adopting the health belief model (HBM) [10,11] perspective. The HBM states that, in response to a threat, an individual’s health behavior is determined by two cognitive processes: how severe one assesses the consequences of a threat to be (ie, threat appraisal) and how efficient and feasible a protection behavior is (ie, coping appraisal) [12].

Applied to the current COVID-19 pandemic, threat appraisal consists first of one’s *perceived susceptibility* or perceived risk for contracting SARS-CoV-2. We expect that if someone perceives themselves to be at risk of COVID-19 infection and related health complications, the individual will be inclined to use the app to assess potential COVID-19 infection risks. *Perceived severity* refers to individuals’ perceptions of the impact of infection for them. Therefore, individuals who assess this risk to their personal health as high will be more inclined to adopt the app.

Behavioral intention is further determined by the *perceived benefits*—in this case, the expected positive consequences of using the COVID-19 app. Individuals who are more convinced of the app’s social (eg, using the app to contribute to knowledge about the viral spread) and individual (eg, being informed of potential infection) benefits would be more willing to use the app. However, in the current debate on tracing apps, some have voiced concerns about the protection of app users’ personal data [3]. These concerns can form *perceived barriers* to adopt the app. Additionally, tensions may occur between infected and healthy individuals [13], which could also present barriers to using the app. By contrast, *cues to action* can stimulate individuals to engage in protective behaviors. Since media coverage of the COVID-19 pandemic is high, we assessed respondents’ perceived exposure to (digital) media content. We expect that the more individuals consult news platforms during the pandemic, the more inclined they will be to use the app.

Users may have various expectations concerning their potential mastery of the app. Individuals’ *self-efficacy* was added to the original HBM [14], which is, in short, one’s belief of having mastered performance of a requisite protective behavior [15]. We therefore expect that individuals’ adoption of the app will be influenced by their belief in their competence to use the app. The HBM is often complemented by factors that relate to the particular behaviors being investigated [16]. We included health conditions that increase respondents’ risk when infected with the virus as an additional factor that may influence behavioral intentions. Finally, we investigate potential differences in gender, age, and education.

## Methods

### Procedure and Sample

Our study was conducted in Belgium, one of the top 15 countries with the greatest number of cumulative confirmed COVID-19 cases (from January to April 2020) [17]. At the time of this study, no contact tracing technology had been implemented in Belgium.

An online survey was administered to respondents, aged 18 to 64 years. The study was approved by the University of Ghent Ethics Committee. The data were collected from April 17 to 19, 2020. The recruitment of respondents was organized by a professional research agency.

Using the statistical program G*Power, the calculation of an *a priori* sample size, with an effect size of 0.1, a desired power value of at least .80, and an alpha score of no greater than .05, returned a recommended minimum sample size of 614 respondents.

A sample of 1500 respondents was recruited with the following eligibility criteria: (a) a resident of Belgium, (b) aged 18-64 years, and (c) speak Dutch. To achieve a heterogeneous sample, we followed a stratified sampling procedure. Based on Belgian federal statistics, we stratified *a priori* the data regarding gender (50.42% male and 49.58% female), age (33.28% between 18-34 years, 32.15% between 35-49 years, and 34.57% between 50-64 years), and educational degree (22.50% with lower secondary education, 40.65% with upper secondary education, and 36.85% with higher education) so that the proportion of the sample’s strata would reflect the Flemish population. In total, 8000 panel members were emailed an invitation to participate, which included a short description of the study. When 1500 respondents were recruited, in accordance with the strata, data collection was truncated. Respondents were not remunerated for their participation but were entered into a contest organized by the agency to win vouchers worth a maximum of 50 euros.

The respondents were informed of study objectives and asked for informed consent. They were then provided with a brief description of the key features of a potential COVID-19 app—the use of Bluetooth or GPS signals to detect proximity, the anonymous disclosure of users’ COVID-19–positive status to other users who have been in their proximity, access to supplementary information, and advice on dealing with COVID-19. This information was based on available explanations from apps that have been developed [18,19] since a COVID-19 app was not available in Belgium at the time of the study. This introduction and the questionnaire were assessed by 3 respondents to check for clarity.

### Measures

We measured HBM constructs following Champion’s recommendations [20]. All answers were on 5-point Likert scales ranging from *disagree* to *agree*. *Perceived susceptibility* was measured with 3 items assessing respondents’ views on how likely a COVID-19 infection would affect them. *Perceived severity* was assessed with 3 items investigating how serious respondents assess the consequences for their health of a COVID-19 infection to be. In total, 6 items measured the *perceived benefits* respondents find in using the COVID-19 app (individual as well as social benefits). Based on current debates about COVID-19 apps, 2 items measured *perceived barriers*. This construct focused on privacy issues raised by the app and how it could contribute to tensions among citizens with a different COVID-19 status. *Cues to action* that would stimulate individuals to use the app concentrated on (online) news consumption during the COVID-19 crisis. This news consumption was measured by asking respondents: “When you think of the news you consult during the corona period (this is the period since the Belgian government announced strict measures on Friday, March 13, 2020), how often do you consult the news through the sources below?”. In line with previous research [21,22], respondents rated the online sources. Answers were recorded using a 5-point scale ranging from *never* to *multiple times a day*. Finally, 3 items were designed to capture *self-efficacy*, which is the respondents’ own assessment of how easy it would be for them to use the app. In addition, the respondents’ gender, age, and education level were asked. Finally, individuals’ COVID-19 personal health risk was assessed by asking if they suffered from one or several health conditions that can be a risk factor when infected with SARS-CoV-2 (ie, heart or lung condition, renal disease, diabetes, cancer, weakened immune system, high blood pressure).

### Data Analysis

We applied structural equation modeling to the collected data using Mplus 8.4 (Muthén & Muthén) to examine the relationships among the HBM constructs [23]. First, we built a measurement model to test whether the observed variables reliably reflect the hypothesized latent variables (ie, intention, perceived susceptibility, perceived severity, perceived benefits, perceived barriers, cues to action, self-efficacy). Thereafter, we examined the relationship between the study variables and our covariates (ie, gender, age, education, COVID-19 personal health risk). Finally, we estimated a structural model with intention to use the COVID-19 app as the outcome.

We evaluated the model fits of the measurement and path models according to several fit indices. Given that the χ^2^ is almost always significant and not an adequate test of the model fit [24,25], we also report the comparative fit index (CFI), root mean square error of approximation (RMSEA), and the standardized root mean square residual (SRMR). The CFI ranges from 0 to 1.00, with a cut-off of .95 or higher indicating that the model provides a good fit [24,26]. RMSEA values below .05 indicate a good model fit [27]. The SRMR is a standardized summary of the average covariance residuals [25]. A relatively good model fit is indicated when the SRMR is less than .08 [26].

## Results

### Descriptive Results

Descriptive statistics of the variables, together with Cronbach alpha values of the constructs, are presented in Table 1. A correlation matrix of the latent variables is presented in Table 2. All items were included in the survey in Dutch and were translated for this paper. Table 3 provides descriptive statistics of the sample, including age, gender, and highest level of education.

**Table 1:** Description of study variables.

Question		Score, mean (SD)	Cronbach alpha
**Behavioral** **intention**			.98
	BI1. I would be willing to use the COVID-19 app.	3.18 (1.41)	
	BI2. I plan to use the COVID-19 app.	3.08 (1.40)	
	BI3. I want to use the COVID-19 app in the future.	3.18 (1.41)	
**Perceived susceptibility**			.74
	PSU1. I am at risk of being infected by the COVID-19 virus.	2.86 (0.95)	
	PSU2. It is likely that I would suffer from the COVID-19 virus.	3.4 (0.99)	
	PSU3. It is possible that I could be infected by the COVID-19 virus.	3.18 (1.07)	
**Perceived severity**			.85
	PSE1. If I were infected by the COVID-19 virus, it would have important health consequences for me.	3.74 (1.02)	
	PSE2. If I were infected by the COVID-19 virus, my health would be severely affected.	3.7 (1.04)	
	PSE3. If I were infected by the COVID-19 virus, my health would be significantly reduced.	3.79 (1.01)	
**Perceived benefits**			.90
	PBE1. The COVID-19 app will offer me the opportunity to contribute to better knowledge about the spread of the virus.	3.49 (1.17)	
	PBE2. With the COVID-19 app, I will collaborate to reduce the spread of the COVID-19 virus.	3.38 (1.23)	
	PBE3. Thanks to the COVID-19 app, I will be more on my guard when I have face-to-face contact.	3.36 (1.23)	
	PBE4. Thanks to the COVID-19 app, I will take more precautions not to spread the COVID-19 virus myself (eg, wash my hands, maintain distance from others [social distancing], limit my outside movements).	3.18 (1.26)	
	PBE5. By using the COVID-19 app, I will help public authorities to combat the COVID-19 virus.	3.45 (1.20)	
	PBE6. The COVID-19 app will allow me to protect myself from the COVID-19 virus.	3.37 (1.17)	
**Perceived barriers**			.60
	PBA1. The COVID-19 app will reduce its users’ privacy.	3.69 (1.11)	
	PBA2. The COVID-19 app will create tensions between individuals who are infected by the COVID-19 virus and those who are not.	3.61 (1.09)	
**Cues to action**			.66
	CTA1. Website of a newspaper, TV or radio station, or magazine.	4.14 (1.82)	
	CTA2. App of a newspaper, TV or radio station, or magazine.	2.89 (2.03)	
	CTA3. News shared on social media (Facebook, YouTube, Twitter, Instagram, etc).	3.68 (1.87)	
	CTA4. News shared through messaging apps (personal messages through WhatsApp, Messenger, etc).	2.99 (1.95)	
	CTA5. Alerts through email and newsletters.	2.94 (1.81)	
**Self-efficacy**			.79
	SE1. I have the knowledge needed to use the COVID-19 app.	3.62 (1.23)	
	SE2. I have the necessary resources to use the COVID-19 app.	3.78 (1.21)	
	SE3. I can get help from others if I experience difficulties using the COVID-19 app.	3.71 (1.14)	

**Table 2:** Correlation matrix of latent variables.

Variable	1	2	3	4	5	6	7
1. Behavioral intention							
2. Perceived susceptibility	.009						
3. Perceived severity	.080^a^	.078^a^					
4. Perceived benefits	.468^a^	.007	.170^a^				
5. Perceived barriers	–.052^b^	.138^a^	.057^b^	.103^a^			
6. Cues to action	.228^a^	.046	.071^a^	.198^a^	.085^a^		
7. Self-efficacy	.285^a^	.068^a^	.023	.205^a^	.196^a^	.211^a^	

^a^*P*<.01.

^b^*P*<.05.

**Table 3:** Characteristics of the study sample.

Characteristic		Study sample (N=1500)	
**Gender, n (%)**			
	Male		756 (50.4)
	Female		744 (49.6)
**Age (years), mean (SD)**		41.58 (13.94)	
	18-34, n (%)		499 (33.3)
	35-49, n (%)		483 (32.2)
	50-65, n (%)		518 (34.5)
**Educational level, n (%)**			
	No diploma or primary or lower secondary education diploma		338 (22.5)
	Secondary education diploma		611 (40.7)
	Higher education diploma		551 (36.7)

In total, 48.70% (n=730) of respondents agreed with the statement that, when launched, they intend to use the app; 20.40% (n=306) disagreed, 10.40% (n=156) somewhat disagreed, 20.50% (n=308) neither disagreed nor agreed, 27.90% (n=418) somewhat agreed, and 20.80% (n=312) agreed that they intended to use the COVID-19 app. No significant differences were found between women (n=356, 47.80%) and men (n=374, 49.50%) in their intention to use the app (χ^2^_1_=0.395, *P*=.53). Comparing the three age categories of respondents resulted in no significant differences in app adoption intentions between 18-34-year-olds (n=234, 46.90%), 35-49-year-olds (n=247, 51.10%), or 50-65-year-olds (n=249, 48.10%) (χ^2^_2_=1.883, *P*=.39). Regarding respondents’ education, individuals with higher education did not significantly differ in their intention to use the app (n=261, 47.4%) from respondents with, at most, secondary education (n=469, 49.4%) (χ^2^_1_=0.588, *P*=.44). Individuals suffering from health conditions that make them more vulnerable to COVID-19 complications did not differ in their intention to use the app (n=243, 50.10%) compared to respondents without health problems (n=487, 48.00%) (χ^2^_1_=0.592, *P*=.44).

### Measurement Model

The measurement model provided a good fit for the data (χ^2^_254_=750.87, *P*<.001; CFI=.976, RMSEA=.036, 90% CI .033-.039, SRMR=.034). All factor loadings were significant and above .44. We subsequently included age, gender, education, and COVID-19 personal health risk as covariates in the analyses and examined the relationships between the covariates and the study variables.

Gender and education were not significantly associated with any of the study variables. Age was significantly related to perceived severity (*β*=.20, *P*<.001), susceptibility (*β*=–.21, *P*<.001), benefits (*β*=–.08, *P*=.003), and self-efficacy (*β*=–.17, *P*<.001). Having a health condition that can be a risk factor when infected with COVID-19 was not significantly related to the model’s constructs. Our structural model has been adjusted for these variables’ influence.

### Structural Model

**Figure 1:**
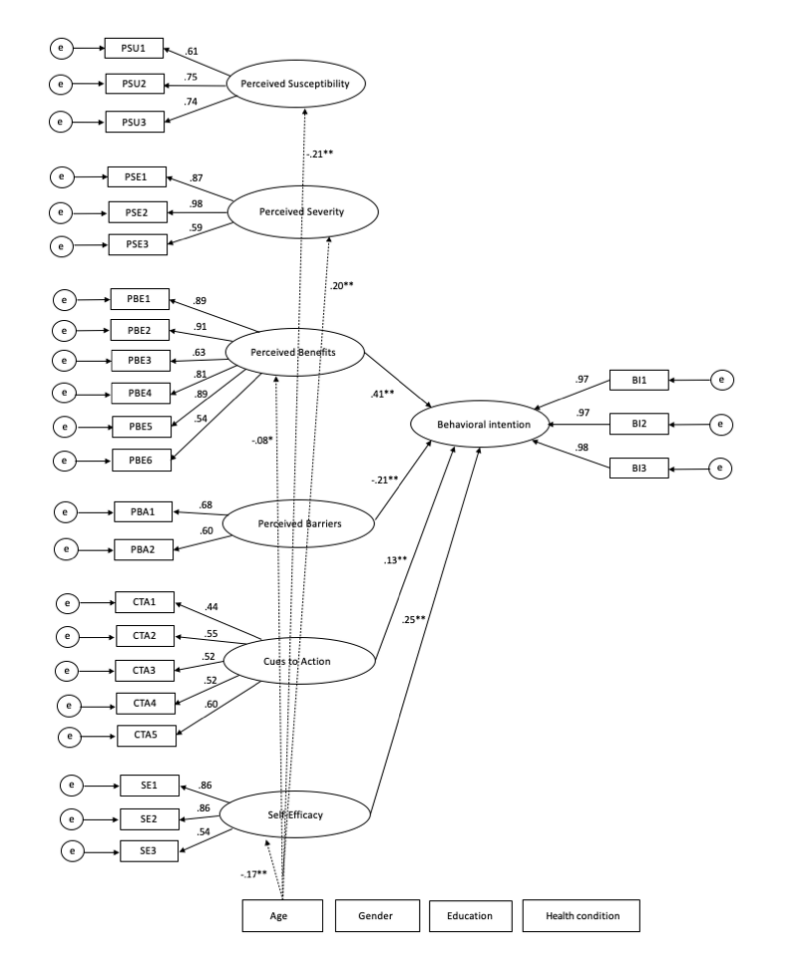
Structural model. Nonsignificant paths are not included. Dashed lines refer to covariates. *P<.01 **P<.001.

The results of the structural model are presented in Figure 1. The results of the fit statistics indicate a good model fit (χ^2^_350_=1070.46, *P*<.001; CFI=.966, RMSEA=.037, 90% CI .035-.040, SRMR=.042).

Our analyses revealed that perceived severity, perceived susceptibility, perceived benefits, perceived barriers, cues to action, and self-efficacy, together with the covariates, explained 32.30% of the variance in intention. The most important predictor of intention was perceived benefits (*β*=.41, *P*<.001), followed by self-efficacy (*β*=.25, *P*<.001) and perceived barriers (*β*=–.21, *P*<.001). Cues to action were significantly related to intention (*β*=.13, *P*<.001). However, perceived severity (*β*=.01, *P*=.95) and perceived susceptibility (*β*=.03, *P*=.38) were not significantly associated with intention.

## Discussion

### Principal Results

In recent months, several countries have implemented or are discussing the integration of a COVID-19 app in their deconfinement plans [28]. Still, questions remain regarding citizens’ motivation to use the app. Epidemiologists state that more than half of the population should use a contact tracing app for it to become effective [29]; in our study sample, almost half intend to use it.

As far as the HBM constructs are concerned, we found that perceived benefits, self-efficacy, perceived barriers, and cues to action were associated with respondents’ intention to adopt the app. However, perceived severity and perceived susceptibility were not. This last finding is consistent with meta-analyses of studies that used the HBM or the related protection motivation theory. These studies showed that, in general, threat appraisal (vulnerability and severity) was least often significantly associated with intention, whereas coping appraisal (perceived benefits and self-efficacy) proved to be more consistently associated with health-related intentions and behaviors [30-32]. This suggests that future research and initiatives to stimulate COVID-19 app uptake should investigate the best ways to enhance perceived benefits and self-efficacy. An optimal strategy proposed by Bandura [33] is to provide individuals with concrete experiences with a target behavior, for instance, through role-play. Offering potential users a clear go-through where they experience the use of the app, the limits of its data processing, and the clarity of the app’s feedback could make the advantages more concrete. Especially because the present study showed a negative relationship between age and self-efficacy, it is important to develop information on the app’s usability that is suitable for all age groups. Moreover, older potential users need to be more convinced of the app’s benefits, as a negative relationship was found between age and perceived benefits.

Based on our findings, individuals’ belief of the gravity of the COVID-19 crisis and their personal vulnerability did not predict app uptake intention. When the threat is assessed as severe and the prevention behavior is complex or not well known, the role of perceived vulnerability may be diluted [32]. This could be the case for a novel COVID-19 app, which could be seen by some respondents as too complex a digital tool to use. Other variables related to app use might be involved. Further research could therefore assess how respondents perceive the ease of use of the app and how app usage can be swiftly integrated in their daily routines.

Another possible reason for the nonsignificance of threat appraisal in terms of adoption intention could be that the government’s stay-at-home order could lead people to think that they are less susceptible to the virus. However, at the time of the survey, the Belgian government’s confinement measures still allowed citizens to go outside for a walk and participate in individual sports and shopping (in grocery stores, supermarkets, and pharmacies). Working from home was mandatory (except for specific sectors and positions). Interpersonal contact was limited to people living under the same roof. Although physical distancing and wearing a mask were advised (but not compulsory), people could be in close proximity to each other and thereby contract the virus; hence, at that stage of the crisis, the app could have been useful. Occasions to be in close proximity with other people were possible but limited. This limited contact with others could have influenced individuals’ threat appraisal and its relation to app uptake intention.

Furthermore, perceived barriers and cues were significantly related to app uptake intention. A perceived barrier for some potential users is their concern about privacy. Especially in a health care context, concerns on the security and confidentiality of data can rise. Privacy advocates have raised concerns about data protection issues related to the implementation of contact tracing apps [34,35]. That is why some contact tracing methods that do not use location data have been proposed [1]. By using data-minimizing solutions, not only are the privacy rights of users being protected but the impact of the app will increase as more people trust and thus install it [2]. Therefore, when developing and launching an app, how individuals’ privacy is protected should be further clarified to potential users. In this respect, citizens’ privacy and other concerns should be further investigated to gain insight into factors that could slow down app uptake.

Cues to action were found to positively correlate with app use intention. In recent months, the media have extensively reported on the pandemic and response measures that have been taken [36]. Additionally, contact tracing apps have been frequently discussed. Although the country where this study was conducted did not implement a COVID-19 app, several strategies such as using traditional contact tracing (through a call center) or a contact tracing app were discussed in mass media and on social media. Our study found a positive relationship between exposure to (online) information and intention to adopt the app. As its effectiveness depends on the app’s uptake, further insight is needed into media coverage on the app’s functionalities and effectiveness. At the same time, it is important to analyze press coverage and online conversations to gain insight into questions that are raised concerning the app’s ethical and legal challenges and how they are addressed. Next to research on how the media report the COVID-19 crisis [37], specific *framing analyses* could be conducted to examine news items and online comments concerning contact tracing apps. Results could inspire governments’ and companies’ app development and communication strategies. In addition, how citizens’ media consumption (specifically, potential changes in media consumption during a crisis period) influences citizens’ attitudes and behavioral intentions toward the app could be investigated.

Because perceived benefits formed the most important factor in relation to app uptake intention, the functionalities and efficacy of the app in controlling COVID-19 should be made clear. Therefore, when launching a COVID-19 tracking app, the importance of tracing contacts and reporting possible exposure to the virus needs to be explained and visualized. Several presentations have been created to concretize the aerosolization of the virus through breathing and could inform on how using a COVID-19 app could map close individual contact that presents a high propensity for infection. To sustain perceived benefits in the long run, supplementary options could be integrated to inform and assist users (eg, including advice on preventing COVID-19–related infection, supplementary resources, and professional assistance). In sum, the app could be further developed as a central hub including detection, advice, and assistance to avoid infection as well as provide users advice during self-isolation [38].

Notwithstanding the value of a contact tracing app, this technology is only one potential instrument. Even with great uptake, some transmissions of the virus (eg, through objects) may not be captured [1]. Therefore, contact tracing needs to be integrated into broader public health interventions, including raising awareness of preventive behaviors and testing [38]. Moreover, the effectiveness of contact tracing apps depends on the general public’s uptake. Uptake by a substantial portion of the population is needed to collect enough data. Therefore, further insight into the predictors of contact tracing app adoption is needed to influence uptake and continued use.

### Limitations

Notwithstanding its results, this study has some limitations. First, although our sample was heterogeneous with regard to age, gender, and educational level in Flanders (ie, the Dutch speaking part of Belgium), the use of convenience samples limits the generalizability of our findings. Furthermore, due to our sampling procedure we may have specifically missed out those who are already disadvantaged and less visible in society due to a lower income level, health status, social status, or migration background. Corroboration of our findings produced by representative data as well as data derived from disadvantaged groups would lend credibility to the findings.

Second, because COVID-19–related apps have not yet been deployed in Belgium (at the time this study was conducted), future research could investigate individuals’ uptake when an app is launched. Additionally, in countries in which a similar app has already been released, determinants of use and, even more importantly, continued use should be investigated. Future research could investigate app uptake (intention) longitudinally to assess citizens’ willingness to use the app and whether changes in threat and coping appraisal occur at different levels of the COVID-19 outbreak and influence intention and behavior.

Third, since we measured intention to use the app based on a general app description, future researchers could use vignettes to describe several concrete options and their combinations to assess how respondents would be willing to adopt the app, depending on specific characteristics.

### Conclusion

Contact tracing apps are being considered by many governments as a crucial part of their lockdown exit strategies during the COVID-19 pandemic. High uptake is crucial for these apps to be efficient in the mitigation of the virus. However, it remains unclear how we can motivate citizens to use these apps. Our results indicate that it is necessary to act on citizens’ perceived self-efficacy and increase the perceived benefits of COVID-19 apps. At the same time, perceived barriers such as privacy concerns have to be overcome. Finally, the media can play an important role in stimulating app uptake by informing citizens about the functions, benefits, and use cases of the app, thereby increasing self-efficacy and perceived benefits.

## Abbreviations

CFI: comparative fit index
HBM: health belief model
RMSEA: root mean square error of approximation
SRMR: standardized root mean square residual
